# Sociodemographic and Digital Education Factors Are Associated to General Health, Coping Strategies, and Academic Outcomes of Undergraduate Students during the Post-Pandemic Period

**DOI:** 10.3390/ejihpe12090093

**Published:** 2022-09-06

**Authors:** Luigi Tinella, Andrea Tinterri, Anna Dipace, Manuela Ladogana, Isabella Loiodice, Andrea Bosco

**Affiliations:** 1 Department of Educational Sciences, Psychology, Communication, University of Bari, 70121 Bari, Italy; 2 Department of Humanities, University of Foggia, 71121 Foggia, Italy; 3 Telematics University IUL, 71121 Foggia, Italy

**Keywords:** education, COVID-19, remote education, well-being, multiple regression

## Abstract

The COVID-19 pandemic suddenly and forever changed the lives of many undergraduate students around the globe, forcing them to switch to online learning while undergoing social confinement within their homes. It is now well-established that this prolonged period of uncertainty impacted students’ well-being, health, and academic achievement. However, how student-related factors, such as coping strategies as well as sociodemographic, contextual, and technological variables, are linked to digital education factors is currently less understood. Using multiple regression analysis, this study investigates the results of an online questionnaire administered to students from two universities in southern Italy, differing in positioning and size, as well as policies and attitude towards digital learning. The results of this study show the positive effects of expertise with digital devices and university digital learning policies on students’ perceived general health. Conversely, isolation and lack of relational connectedness negatively impacted students’ health. Furthermore, this study highlights the role of different coping strategies, demonstrating that active forms of coping have a positive effect on students’ health, whereas avoidance strategies have the opposite effect. Taken together, this study provides crucial links between the many factors influencing students’ experience with online learning and health, and provides useful indications to promote the uptake of and adaptation to online learning.

## 1. Introduction

The emergence of the coronavirus disease (COVID-19) saw the imposing of several extraordinary measures to limit the spread of the Severe Acute Respiratory Syndrome Coronavirus 2 (SARS-CoV-2 virus). Strategies such as lockdowns have been promoted all over the world, with documented effects on people’s mental and physical health [1]. In particular, the “stay-at-home” strategy has promoted feelings of uncertainty, present and future instability, insecurity, as well as reduced autonomy [2,3,4]. Among the restrictive measures, education institutions around the world in early 2020 cancelled or postponed research activities, conferences, workshops, etc. Rapidly, courses have also been converted from face-to-face to online education, with negative consequences on students’ health [5], from primary school to higher education institutes. Prolonged confinement measures determining social isolation, fear of infection, space constrictions, boredom, and frustration caused an increase in both depressive and post-traumatic symptoms among the population [6,7]. Therefore, the education system has been considerably distressed by these restrictive measures [8,9], which affected more than 6 billion students from 190 nations, of which 24 million were at serious risk of drop-out [10]. In Italy, starting from March 2020, all educational and related activities, at all levels, were suspended. Subsequently, further restrictive measures were introduced from October 2020 to limit the diffusion of SARS-CoV-2. The restart of activities at Italian universities was scheduled for January 2021. To date, vaccinations have proven to be an effective strategy reducing the spread of the COVID-19 infection. Despite this, the pandemic notably affected various aspects of teaching and learning [11,12].

Considering the above, several studies highlighted the stressful effects of the academic burden, as well as the lack of relaxation time among undergraduate students [13]. During the outbreaks, both students and teachers complained of more difficulties in staying focused and being productive [14]. Sahu [5] documented that the interruption and the postponement of exams and graduation sessions, the fear that the contagion could negatively affect performance in the exam, and worries about future careers were frequently reported, not only by students but also by whole academic communities, promoting feelings of fear, stress, and anxiety. The school closures increased the perceived stress and sedentary behaviours, with negative effects on psychological well-being [15]. Moreover, a cross-national survey involving emerging adults revealed that the reduction in physical exercise was related to a lower mood [16]. In the study of Tang et al. [17], it was demonstrated that being senior students and being about to graduate were factors associated to sleep disorders and an increased risk of the development of both Depression and Post Traumatic Stress Disorders.

The massive use of online learning also has had negative effects on students’ well-being, and has revealed shortages in the higher education system [18]. Despite the potential of online learning (and, more generally, of digital education [19]) in improving students’ academic outcomes being well-recognised [20], several studies have highlighted its negative effects from a psychological viewpoint; among others, we can see increased anxiety due to the lack of physical interaction with peers [21], or more broadly to the difficulty with adapting [22]. Moreover, the digital divide, that is, the disparities in accessing and using information and communication technologies [23], as a form of socio-economic inequality, also influenced students’ health and academic performance during the pandemic. By comparing some regional inequalities, the pandemic seems to have increased the gaps in access to a formal education and learning opportunities, highlighting the relationships between poverty and psychological discomfort [24,25,26].

Several studies highlighted that sociodemographic factors have a great impact on the effectiveness of online learning [27] and on students’ quality of life [28]. It seems that age, the study program, and years of education are significant determinants of the effectiveness of remote learning [27]. During the pandemic, indeed, demographic characteristics such as gender and the presence of cohabitants played an important role. It has been demonstrated that anxiety symptoms among students are associated with the area of residence, the parental income, and whether the subject lives with their parents [29]. Female participants and those students living alone were at a greater risk of negative mental health consequences [19,30]. All these aspects contributed to increased anxiety among students and promoted the fear of failing the academic year [31].

Already before the pandemic, some contextual (i.e., university settings, culture, rule, regulations) and technical issues (i.e., familiarity with technology) had been identified as the most important challenges in the implementation of online learning for instructors [32,33]. These factors also challenged lecturers during the pandemic, and this indirectly created some stress among students [19]. The type of course attended has also been identified as an important variable influencing students’ academic outcomes throughout online learning. For example, online teaching practices may have more serious implications for the academic outcomes of students attending courses requiring practical training [34] than those in which training in practical skills is not required [35]. Moreover, recent research has highlighted that the integration of credible social media use in online learning may attenuate the negative consequences of the COVID-19 pandemic from the perspective of both teachers and students [36]. The online learning-related difficulties encountered during the pandemic have been classified by Baticulon et al. [37] into personal, community, technological, familiar, and institutional categories. Among these, the lack of experience with technological devices, the weakness of online teaching infrastructures, technical problems with devices or with the internet connection, and the difficulties in finding a quiet environment in which to follow the lesson represent the major challenges in both synchronous and asynchronous online education [38,39].

As can be noted, sociodemographic, contextual, and technological factors play a crucial role in influencing both the students’ academic outcomes and their general health in situations of online learning. Despite the great interest, little is known about the impact of changes in learning modalities on undergraduate students’ mental health and academic performance. In their study, the authors of [12] found no significant differences in students’ psychological distress between the face-to-face (f2f) modality and online learning. Moreover, while some studies highlighted students’ comparative preference for online learning over f2f [40], others pointed out their negative opinions of remote learning due to technical problems [41]. Anyway, the relationship between online learning and students’ health and performance seems to have been moderated by the adopted coping strategies.

In studying the effects of sociodemographic, contextual, and technological factors on undergraduate students’ health and performance, several studies focused on students’ coping strategies as determinants of the adaptation to online learning. In the study of Savitsky et al. [42], positive coping strategies (i.e., humour) were found to be associated with positive attitudes (i.e., low anxiety levels). More recently, Rahmat et al. [43] found that positive attitudes toward online learning (or acceptance) significantly correlated with the use of positive coping strategies (e.g., planning, positive reframing, active coping), while negative attitudes were significantly associated with negative strategies (e.g., self-blame, denial, substance use).

Despite the above, little is known about the long-term effects that the sociodemographic and technological variables of students’ lives during the pandemic period have had on their general health, the adoption of coping strategies, and the compliance to remote learning. More generally, it is unclear whether and how the above-mentioned characteristics (i.e., sociodemographic and technological) may have affected students’ perception of the quality of online learning.

The present study aimed to investigate the long-term effects of sociodemographic factors (i.e., gender, number of cohabitants), contextual variables (e.g., the university policy regarding digital education technological aspects (i.e., quality of internet connection, expertise, hours of device use), and perceived loneliness on students’ general health, coping strategies, the perceived quality of online learning, and their compliance to it. In particular, the comparison between the answers given by students from two universities (Foggia and Bari) should help us to understand if there is an influence of the policies adopted by the universities with respect to digital education strategies (such as the practically unrestricted use of blended approaches, even in the post-pandemic period (in Foggia), compared to the more restrictive model, with a stronger aspiration to bringing back all didactic activities). A secondary aim was to evaluate whether the different coping strategies used in the slow return to normality in the post-pandemic period could predict the students’ self-reported general health. To this end, the present study was conducted to better understand the relationship between the student’s health, their sociodemographic characteristics, status variables, and the efficiency of online learning during the pandemic. Among the sociodemographic predictors, both gender and the number of cohabitants were expected to significantly predict students’ health [44] and the perceived quality of online learning [27], respectively. A negative effect of perceived loneliness was hypothesised on both the compliance and the health of students. On the contrary, technological variables were expected to positively predict both the studied outcomes [41]. Finally, it was hypothesised that the adopted coping strategies could predict students’ general health.

## 2. Materials and Methods

### 2.1. Participants

Six-hundred and forty-two undergraduate students aged from 19 to 68 (85.6% females) completed the whole questionnaire in the survey. All participants were included in the final sample. Out of the total sample, 432 students were recruited from the University of Foggia, Apulia (Italy), and 210 attended a university course at the University of Bari, Apulia (Italy). Participants were required to: (a) have Italian as their native language, and (b) be currently attending a university course at the University of Foggia or the University of Bari, Apulia (Italy). The mean age was 24.6 years (SD = 6.50) for students from the University of Foggia and 21.9 (SD = 3.68) for students from the University of Bari. Considering the total sample, 499 participants (77.8%) attended a university course in Pedagogical and Psychological Sciences, 57 participants (8.8%) attended a university course in Medical Sciences, 55 participants (8.6%) a university course in Literature and Philology, and 31 participants were students of Financial and Political Sciences (4.8%). Descriptive statistics are presented in Table 1.

All participants, blind to the hypothesis of the study, were volunteers. They were enrolled and completed the online survey between October 2021 and January 2022. This research was compiled following the tenets of the Declaration of Helsinki, and the ethical committees of both the involved institutions approved the study protocol. All participants signed their informed consent before enrolment in the study.

### 2.2. Materials and Procedures

The participants were recruited via social media, including the universities’ newsletters, as well as through the help of proxy informants (undergraduate students). Interested participants were provided a link to an online survey hosted by SurveyMonkey^®^, which presented details on the study, the consent form, questions on eligibility, questions related to participants’ socio-demographic characteristics (e.g., age, gender, possession of a personal computer, quality of the internet connection at home, number of cohabitants, etc.), information on the academic outcomes (number of attended courses, number of completed courses), and the below-described questionnaires. The survey took approximately 20 min to complete.

#### 2.2.1. Perceived Loneliness (UCLA LS 3)

The UCLA Loneliness Scale Version 3 [45] was administered as a measure of subjective feelings of loneliness and social isolation. The scale is composed of 20 items (e.g., “How often do you feel that you are no longer close to anyone?”) and three factors (i.e., Isolation, Relational Connectedness, and Trait Loneliness). For each item participants were required to rate the statement on a scale from 1 (never) to 4 (often). Inverse scoring was adopted for negative items. The instrument showed acceptable reliability in its scales (Isolation: α = 0.81; Relational Connectedness: α = 0.75; Trait Loneliness: α = 0.60).

#### 2.2.2. Coping Strategies (COPE–NVI 25)

The 25-item version of the Coping Orientations to Problem Experienced (COPE; [46]) is a self-reported questionnaire measuring several coping strategies. It is composed of 25 items describing five main coping mechanisms (i.e., Orientation to Problem—OT; Positive Attitudes—PA; Transcendental Orientation—TO; Social Support—SS; Avoiding Strategies—AS) and for each item respondents were required to report how often they are engaged with that strategy on a Likert scale ranging from 1 (“usually I don’t”) to 4 (“I almost always do”). Reliability coefficients of scales varied from acceptable to excellent (SE: α = 0.60; OT: α = 0.93; AP: α = 0.65; SS: α = 0.81; OP: α = 0.68).

#### 2.2.3. General Health Questionnaire (GHQ-12)

The General Health Questionnaire (GHQ-12; [47,48,49]) was administered to assess participants’ current mental health. This brief, simple, and easy to complete tool is composed of 12 items asking for a report of how often the respondents experienced the listed feelings and symptoms across the previous two weeks on a Likert scale ranging from 1 (“never”) to 4 (“almost every day”). The scale showed good reliability (Cronbach’s α = 0.89).

#### 2.2.4. Perceived Quality of the Online Learning and Resilience to the Online Learning

Two ad-hoc groups of questions have been developed to assess, respectively, the respondents’ perceived Quality of Online Learning (QOL) and their own Resilience to Online Learning (ROL). For each of these constructs, three questions have been developed. For each item, respondents were required to report on their experiences during online university courses on a Likert scale ranging from 1 (“Absolutely false”) to 7 (“Absolutely true”). Both these scales showed acceptable reliability (i.e., QOL: α = 0.77; ROL: α = 0.66).

### 2.3. Data Analysis

Independent samples *t*-tests were performed controlling for differences in self-reported measures between both the university campuses. Bivariate correlation coefficients and related *p*-values were calculated between all the employed variables. To achieve the aims of the present study, a series of multiple regression models was performed using the Jamovi Software [50], considering (a) university campus membership (university policy regarding digital education) and gender group as independent factors, (b) the number of cohabitants (N-C), the quality of the internet connection at home (Q-C), the expertise in the use of electronic devices (EXP), the sum of daily hours of use of devices both during the pandemic period (H-pan) and before the pandemic period (H-pre), and the factors of the loneliness scale (i.e., Isolation; Relational Connectedness; Trait Loneliness) as independent continuous variables. For each of the dependent variables (i.e., General Health, Cope-AS; Cope-TO; Cope-PA; Cope-SS; Cope-OP; QOL; ROL; Number of Attended Courses—AC, and Number of Completed Courses—CC) a multiple regression model was estimated by considering the same set of predictors. Finally, the last regression model was performed to estimate the effects of coping strategies (as predictors) on General Health. To assess the goodness of fit of all the models, we used the total R^2^. Only those models in which predictors explained more than 10% (i.e., R^2^ > 0.10) of the outcome’s variance have been retained and are commented on in the discussion. Following Cooper (2019; page 92) [51], we focused on models with a certain degree of explained variance in order to highlight those effects that represent a reasonable overlap between the studied variables. On the other hand, by commenting on the significant effects of predictors in models with a very low explained variance (e.g., R^2^ < 10%), we would have overlooked the fact that more than 90% of the outcome’s variance is due to other unobserved variables [51]. The value of *p* was set to 0.05 for the calculation of statistical significance.

## 3. Results

### 3.1. Descriptive Statistics and Preliminary Analyses

Means and standard deviations for the variables employed in the study are shown in Table 1. Descriptive statistics are reported separately for both university and gender groups. Correlation coefficients are shown in Supplementary Table S1. Age was significantly negatively correlated with H-pre (r = −0.151; *p* < 0.001), ROL (r = −0.179; *p* < 0.001), and TL (r = −0.110; *p* < 0.001), while it was positively with OT coping strategy (r = 0.201; *p* < 0.001), perceived QOL (r = 0.155; *p* < 0.001), and GHQ (r = 0.171; *p* < 0.05). GHQ was significantly positively correlated with EXP (r = 0.126; *p* < 0.001), the coping strategy of OP (r = 342; *p* < 0.001), and perceived QOL (r = 162; *p* < 0.001), while it was negatively correlated with the loneliness scales (Isolation: r = −0.546; *p* < 0.001; Relational Connectedness: r = −0.459; *p* < 0.001; Trait Loneliness: r = −0.392; *p* < 0.001), the avoiding coping strategy (r = −0.378; *p* < 0.001), and the Number of Attended Courses (r = −0.212; *p* < 0.001). The independent samples t-test revealed significant differences between the two university groups in the measures N-pre (Welch’s t (621) = −6.630; *p* < 0.001), EXP (t (631) = −2.0; *p* < 0.05), TO coping strategy (Welch’s t (556) = −9.11; *p* < 0.001), GHQ (t (640) = −4.44; *p* < 0.001), and perceived QOL (Welch’s t (514.8) = −6.58; *p* < 0.001). Students from the University of Foggia reported higher values in all these measures than students from the University of Bari. On the other hand, students from the University of Bari reported higher scores of Isolation (t (640) = 4.23; *p* < 0.001), Trait Loneliness (t (640) = 5.17; *p* < 0.001), coping strategy of social support (t (640) = 2.4; *p* < 0.05), and ROL (t (640) = 6.0; *p* < 0.001) than students from the University of Foggia.

### 3.2. Multiple Regression Analysis

The first multiple regression model was performed considering the university’s policy on digital education, the gender group, N-C, Q-C, EXP, H-pand, H-pre, and the factors of the loneliness scale (i.e., Isolation; Relational Connectedness; Trait Loneliness) as independent variables and the GHQ as the outcome. The set of predictors globally explained 35% of the outcome’s variance (Adj. R^2^ = 0.353). Significant results emerged for the positive effects of the universities’ policies towards digital education (β = 1.08; *p* < 0.05), the gender group (β = 1.30; *p* < 0.05) and EXP (β = 0.66; *p* < 0.05), and for the negative effects of Isolation (β = −0.87; *p* < 0.001) and Relational Connectedness (β = −0.54; *p* < 0.001). Membership to both the university campus of Foggia and the group of male students significantly and positively predicted higher GHQ scores. In the same way to the increase in the EXP, an increase in the GHQ score was observed. On the other hand, with an increase in the reported scores of Isolation and Relational Connectedness, a decrease in the GHQ score was predicted. This model has been retained for the discussion. Table 2 shows significant and non-significant effects for this model.

The second model was employed considering the same set of predictors as in the previous model, and the TO coping strategy as the outcome. Predictors globally explained 9.8% of the total variance (Adj. R^2^ = 0.098). As in the previous model, significant results emerged for the positive effects of the university’s policy towards digital education (β = 2.15; *p* < 0.001) and the negative effects of relational connectedness (β = −0.15; *p* < 0.05). The model has not been considered in this discussion.

The third model was conducted considering the avoiding coping strategy as the outcome and the above-mentioned set of independent predictors. The model explained about 9% of the outcome’s variance (Adj. R^2^ = 0.088). Significant results emerged for the effects of the university’s policy towards digital education (β = 0.38; *p* < 0.05), favouring students from the University of Foggia, and for the positive effects of Isolation and Trait Loneliness (Isolation: (β = 0.18; *p* < 0.001); Trait Loneliness: (β = 0.15; *p* < 0.05)). The higher the scores in these two loneliness scales, the higher the likelihood of resorting to the AS coping strategy. The model was not retained for the discussion.

The fourth model was performed considering the coping strategy of positive attitude as the outcome and with the same predictors as in the previous model. The predictors globally explained 8% of the outcome’s variance (Adj. R^2^ = 0.080). Significant results emerged for the negative effects of both Relational Connectedness (β = −0.16; *p* < 0.05) and Trait Loneliness (β = −0.23; *p* < 0.01). The higher the score reported in these two scales, the lower the likelihood of resorting to positive attitude as a coping strategy. The model was not retained for the discussion.

The fifth model was performed on the social support coping strategy. The set of predictors globally explained 16% of the variance (Adj. R^2^ = 0.160). Significant results emerged for the positive effect of Isolation (isolation: β = 0.34; *p* < 0.05)) and the negative effects of the other two loneliness scales (Relational Connectedness: (β = −0.60; *p* < 0.001); Trait Loneliness: (β = −0.91; *p* < 0.05)). The use of social support as a coping strategy was positively predicted by Isolation and negatively by predicted by Loneliness and Relational Connectedness. The model was retained and is discussed below. Table 3 shows the significant and non-significant effects for this model.

The sixth model was performed considering the OP coping strategy as outcome. The model explained 6.9% of the outcome’s variance (Adj. R^2^ = 0.06). Significant results emerged for the effects of university policy towards digital education (β = −0.55; *p* < 0.05), EXP (β = 0.34; *p* < 0.01), and Trait Loneliness (β = −0.19; *p* < 0.05). Membership at the University of Bari as well as EXP positively predicted the resort to this kind of coping strategy, while with an increase in Trait Loneliness, a decrease in the resort to the OP strategy was observed.

The seventh model was performed on the measure of ROL as outcome. The set of predictors globally explained 5% of the global variance (Adj. R^2^ = 0.05). Significant results emerged for the negative effects of the university policy towards digital education (β = −3.17; *p* < 0.001). Membership at the University of Bari significantly predicted a higher ROL. The model was not retained for the discussion.

The eighth model was conducted considering the measure of QOL as outcome. The set of predictors globally explained more than 12% of the outcome’s variance (Adj. R^2^ = 0.122). Significant results emerged for the effects of the university policy towards digital education (β = 2.83; *p* < 0.001), Q-C (β = 1.03; *p* < 0.001), EXP (β = 0.58; *p* < 0.05), and Isolation (β = −0.28; *p* < 0.05). The policies adopted by the University of Foggia significantly and positively predicted the perceived QOL, which was also positively predicted by EXP and Q-C, and negatively by Isolation. The model was retained for the discussion. Table 4 shows the significant and non-significant effects for this model.

The ninth model was conducted on the number of attended courses. The set of predictors explained less than 0.1% of the outcome’s variance (Adj. R^2^ = 0.001). No significant results emerged. For this question, the model was not retained in the discussion.

The tenth model was performed on the number of completed courses. The model globally explained 4.8% of the variance (Adj. R^2^ = 0.048). A significant result emerged for the effect of university campus membership (β = −0.04; *p* < 0.05), indicating that membership at the University of Foggia positively predicted the number of completed courses. The model was not retained for the discussion.

The last model was performed considering the different coping strategies as predictors and GHQ as the outcome. The model globally explained 26% of the variance (Adj. R^2^ = 0.255). Significant effects were found for the negative effects of AS coping strategy (β = −0.08; *p* < 0.001), and for the positive effects of the TO strategy (β = 0.21; *p* < 0.001), the PA strategy (β = 0.50; *p* < 0.001), and the OP strategy (β = 0.26; *p* < 0.05). With an increased likelihood of resorting to the AS strategy, a reduction in GHQ was observed, while the higher the likelihood of resorting to PA, OP, and TO strategies, the higher the observed GHQ score. The model was retained for the discussion. Table 5 shows the significant and non-significant effects for this model.

## 4. Discussion

The present study aimed to investigate the effects of sociodemographic variables (e.g., gender group, number of cohabitants), contextual factors (e.g., the university policy towards digital learning), and technological variables (i.e., quality of internet connection, expertise in the use of computers) on the measures of general health, coping strategies, perceived quality of learning and compliance to digital learning in a sample of undergraduate students during the pandemic.

Sociodemographic, contextual, and technological aspects showed effects on students’ health. Gender, the university’s policy towards digital learning, and expertise in the use of electronic devices significantly and positively predicted students’ self-perceived general health. On the other hand, students’ health was negatively predicted by both isolation and the lack of relational connectedness. Considering the coping strategies, social support was positively predicted by isolation and negatively by both relational connectedness and trait loneliness. The quality of internet connection, expertise in the use of electronic devices, and membership to the university campus of Foggia positively predicted the perceived quality of online learning, which was negatively affected by isolation.

A further aim of the study was to investigate the effects of coping strategies on students’ health. The results show the positive effects of such strategies as Transcendent Orientation, Positive Attitude, and Orientation to Problem, and the negative effects of the Avoiding coping Strategy, on measures of general health.

Among the regression models, only those that explained a certain degree of the outcome’s variance (i.e., R^2^ > 10%) have been considered for the discussion.

### 4.1. General Health

The first model performed on student’s general health showed the significant and positive effects of gender group, university policies toward digital education, and expertise in the use of electronic devices, and the negative effects of isolation and a lack of connectedness. In particular, being part of both the male group of students and the University of Foggia predicted a higher self-reported level of general health. The higher level of health declared by males is in line with the results of previous works, which demonstrated that male students tend to report a lower level of distress, and supports those studies that showed that female students are exposed to a greater risk of developing negative health consequences [19,30] and derive higher benefits from university counselling interventions than males [52]. General health was also significantly predicted by the university’s policy toward digital education. Membership to the University of Foggia positively predicted the score in the general health questionnaire. As noted above, strategies of promoting and providing a shift towards online education differed among the considered universities. While the University of Foggia struggled to allow the rapid creation and accessibility of asynchronous contents (i.e., pre-registered lessons), only synchronous courses were available at the University of Bari. This result shows that the use of blended approaches (e.g., synchronous and asynchronous) to online education has a positive influence on students’ health. The result seems to suggest that blended approaches are preferable to more restrictive f2f approaches.

The results of this study show the positive effects of expertise on students’ health: the higher the self-declared expertise in using electronic devices, the higher the self-reported general health. This result is not surprising, and emerged mainly as a consequence of the positive effects of the technical knowledge consolidated over time while undergoing online learning through personal technological devices. The fact that the employment of digital technologies improves students’ performance has been previously proven [19]. During the pandemic period, only remote learning was available, so the desirable effects of knowing how to properly use technological devices clearly constituted a sort of advantage for the more expert students, who also declared a higher level of general health.

On the other hand, the results show that a decrease in general health was observed, coupled with an increase in measures of isolation and lack of relational connectedness. As reported in the introduction, the confinement measures caused psychological distress in the general population by affecting both the perceived social and emotional loneliness [53]. For undergraduate students, several aspects, alone or connected, may have had an influence on the lack of social connectedness and on the sense of isolation; for example, the distance between the place of residence and those of relatives and friends [29,30], or the lack of physical contact with persons other than cohabitants [54]. Finally, the results of the longitudinal study by Latikka et al. [55] on the general population show that distress increased during the pandemic only among lonely individuals, and that it was positively predicted by a lower engagement in social media identity bubbles.

The second model, performed on general health, showed that four out of five coping strategies significantly predicted students’ general health. While Transcendent Orientation, Positive Attitude, and Orientation to Problem predicted higher levels of general health, the avoiding coping strategy showed negative effects. The results support those of previous studies, which showed that positive coping strategies are important variables able to moderate the relationships between pandemic-related difficulties (both personal and academic) and the self-reported level of health [42,43].

### 4.2. Social Support as Coping Strategy

Among the considered coping strategies, social support was the only one that had a quantity of explained variance greater that 10%. This coping strategy was significantly and positively affected by isolation, and negatively affected by both the lack of relational connectedness and trait loneliness. In other words, while isolation motivated students to engage in social support as a coping strategy to face pandemic-related difficulties, trait loneliness and the lack of social connectedness on the other hand seem to have discouraged engagement in this coping strategy. This result is quite novel, and suggests the important role played by perceived loneliness in using social support as a coping strategy during the pandemic. In the study of Godfrey [56], social support and social connection emerged as important factors underlying students’ well-being during the return to school, and these effects were significantly more likely among female than male students. Further studies are needed to clarify whether the relationship between the individual psychological well-being and the perceived university context is mediated by social support as a coping strategy.

### 4.3. Perceived Quality of Online Learning

The perceived quality of the provided online learning was significantly and positively predicted by the quality of internet connection, expertise in the use of electronic devices, and the policy adopted by the university (favouring the University of Foggia), while negatively affected by isolation. These results suggest that technological and contextual factors play an important role in the students’ perceived quality of learning over and above their sociodemographic characteristics. First, as in the case of general health, expertise in using technological devices positively influenced the perceived quality of provided learning, confirming the results of previous studies [19]. Together with the positive effects of expertise on measures of health, this result also seems to suggest that expertise in the use of technological devises exerts positive effects on the perceived quality of remote learning, and this in turn promotes a higher level of general health. Further research may address the verification of such a hypothesis. In the same way, this rationale may explain the positive result found for the effect of the quality of internet connection.

Second, university policies regarding digital education may have positively influenced the perceived quality of learning due to important differences in the promotion of online learning by the two universities. Students at the University of Foggia may have perceived higher quality as a consequence of both a smaller (and more easily managed) university community and the higher accessibility of academic content for students. Unfortunately, to the best of our knowledge, no studies have previously measured these variables while comparing groups of students from universities of different sizes.

Third, isolation showed negative effects on the perceived quality of learning over and above technical and demographic factors. This result confirms those of previous studies, which highlighted the crucial role of social isolation in various aspects of students’ life before and during the pandemic [44,54]. Considered together with the negative effects of isolation on measures of general health, it is possible to conclude that isolation may exert negative effects on general health at least partially by negatively influencing the perceived quality of the online learning.

## 5. Conclusions

Overall, this study presents convergent evidence on the effects that sociodemographic, contextual, and technological variables have had on measures of well-being, coping strategies, and perceived quality of online learning among undergraduate students. It has been shown that gender, the university policy towards digital education, expertise in device use, internet connection, isolation, trait loneliness and the lack of relational connectedness are significantly associated to health, the coping strategies used, and the perceived quality of learning. The study presents some important limitations, such as the lack of a sample balanced by gender, the lack of measures for successfully passed exams, as well as the lack of a formal longitudinal design comparing measures taken during vs. after the pandemic period. Moreover, the results obtained on the effect of university policy on digital education deserve more in-depth explanation. The two universities, a little less than 150 km apart, present some important differences that should be noted. First, they host different numbers of students—around 45,000 for the University of Bari and 12,000 for the University of Foggia. Second, the University of Bari was founded in 1925, and it is located almost entirely in a metropolitan area of the city of Bari. The University of Foggia, on the other hand, was founded in 1999, and is located in the city of Foggia, which is a small city. It cannot be ruled out that results presented here may have been influenced by the university communities’ sizes, besides the policies they adopt towards digital education. For example, the size of the university community may have affected the student’s perceived sense of belonging to the community, with negative consequences on psychological health. Finally, the study involved participants from diverse university courses. Since the differential effect of digital learning on the academic outcomes of students attending diverse courses has been previously documented, the type of course may have influenced the obtained results. Further research is required to clarify this, as the impact of digital learning on students’ performance and well-being seems to vary for different university courses. Moreover, those students who experience more serious effects on their academic performance may later encounter greater difficulties in their professional activities and career [34].

Notwithstanding these limitations, the study adds value to the literature by addressing together the student-related variables (i.e., sociodemographic, contextual, and technological variables), the adopted coping strategies, and general health in the context of online learning. These results may be useful in planning interventions promoting the adaptation to online learning. This dimension may be key to returning to this modality in the case of future confinement. Finally, these results may be of great interest for professionals of psychological health in the university context, suggesting tailored interventions [57,58] based on sources of vulnerability (i.e., individual, technical, and contextual) that could affect the students’ mental and physical health, as well as the efficiency of the psychological counselling intervention itself.

## Figures and Tables

**Table 1:** Descriptive statistics divided by university membership and gender groups. Mean (M) and standard deviation (SD) are shown for all the employed variables.

	Bari (N = 210)				Foggia (N = 432)			
	Females (N = 175)		Males (N = 35)		Females (N = 374)		Males (N = 58)	
	M	SD	M	SD	M	SD	M	SD
**Age**	21.8	3.59	22.4	4.10	24.3	6.18	25.7	7.36
**Number of Cohabitants (N-C)**	3.05	1.03	2.97	1.27	3.14	1.50	3.16	2.11
**Quality of Internet Connection (Q-C)**	3.89	0.896	3.77	0.942	4.00	0.855	3.74	1.03
**Expertise in the Use of Devices (EXP)**	3.54	0.828	3.60	0.946	3.70	0.913	3.69	0.863
**Use of Devices during the Pandemic (h-pand)**	8.92	3.07	8.31	3.33	8.72	4.71	7.91	3.89
**Use of Devices before the Pandemic (h-pre)**	5.97	2.93	5.94	3.58	7.21	5.33	6.05	3.28
**Number of Attended Courses (AC)**	1.84	0.920	2.40	0.894	1.67	0.861	1.20	0.447
**Number of Completed Courses (CC)**	2.59	0.615	2.40	0.894	2.37	0.885	2.60	0.548
**Isolation (UCLA ISO)**	10.4	2.47	10.1	2.41	9.50	2.66	9.14	2.55
**Rel. Connectedness (UCLA RC)**	8.40	2.59	8.20	2.25	8.00	2.47	8.45	2.65
**Trait Loneliness (UCLA TL)**	6.76	1.75	6.83	1.65	5.98	1.72	6.31	1.52
**Avoiding Strategy (COPE AS)**	7.99	2.27	8.51	2.16	8.20	2.30	8.09	1.94
**Transcendent Orientation (COPE TO)**	5.63	2.58	6.06	3.11	8.05	3.68	8.10	4.32
**Positive Attitude (COPE PA)**	17.3	3.24	17.2	3.06	17.5	3.03	17.1	2.81
**Social Support (COPE SS)**	13.4	3.51	12.9	3.46	12.7	3.36	11.9	3.16
**Orientation to Problem (COPE OP)**	14.6	3.01	14.1	2.42	14.3	2.56	13.8	2.77
**General Health (GHQ)**	31.0	6.61	32.5	5.53	33.5	6.16	35.0	6.90
**Resilience to Online Learning (ROL)**	14.0	6.70	11.5	6.29	9.93	6.26	13.0	6.36
**Quality of Online Learning (QOL)**	10.6	4.92	9.77	5.01	13.5	6.43	12.9	5.43

**Table 2:** Multiple regression model performed on General Health as an outcome. *: *p* < 0.05; ***: *p* < 0.001.

	1	SE	t	*p*
University’s policies	10.788	0.4730	2.281	0.023 *
Gender	13.042	0.6087	2.143	0.033 *
N-C	0.0672	0.1550	0.434	0.665
Q-C	0.4525	0.2509	1.803	0.072
EXP	0.6584	0.2545	2.587	0.010 *
H-pand	−0.0672	0.0705	−0.952	0.341
H-pre	−0.0141	0.0662	−0.213	0.832
UCLA (ISO)	−0.8735	0.1092	−8.002	<0.001 ***
UCLA (RC)	−0.5401	0.1138	−4.748	<0.001 ***
UCLA (TL)	−0.2295	0.1549	−1.482	0.139
R^2^	0.364			
Adj. R^2^	0.353			

**Table 3:** Multiple regression model performed on social support as an outcome. *: *p* < 0.05; ***: *p* < 0.001.

	β	SE	t	*p*
University’s policies	−0.4636	0.2847	−1.628	0.104
Gender	−0.5672	0.3663	−1.548	0.122
N-C	0.0968	0.0933	1.038	0.300
Q-C	−0.1615	0.1510	−1.069	0.285
EXP	−0.2928	0.1532	−1.912	0.056
H-pand	0.0137	0.0425	0.322	0.747
H-pre	−0.0382	0.0399	−0.959	0.338
UCLA (ISO)	0.3375	0.0657	5.138	<0.001 ***
UCLA (RC)	−0.6090	0.0685	−8.896	<0.001 ***
UCLA (TL)	−0.1914	0.0932	−2.054	0.040 *
R^2^	0.174			
Adj. R^2^	0.160			

**Table 4:** Multiple regression model performed on perceived Quality of Online Learning (QOL) as outcome. *: *p* < 0.05; ***: *p* < 0.001.

	β	SE	t	*p*
University’s policies	28.303	0.5090	5.560	<0.001 ***
Gender	−0.6679	0.6550	−1.020	0.308
N-C	0.0308	0.1668	0.185	0.853
Q-C	1.324	0.2701	3.823	<0.001 ***
EXP	0.5877	0.2739	2.146	0.032 *
H-pand	−0.0626	0.0759	−0.825	0.410
H-pre	−0.0936	0.0713	−1.313	0.190
UCLA (ISO)	−0.2873	0.1175	−2.446	0.015 *
UCLA (RC)	−0.0262	0.1224	−0.214	0.831
UCLA (TL)	0.0961	0.1667	0.577	0.564
R^2^	0.137			
Adj. R^2^	0.122			

**Table 5:** Multiple regression model performed on General Health as outcome. *: *p* < 0.05; ***: *p* < 0.001.

	β	SE	t	*p*
COPE (AS)	−0.8959	0.1023	−8.755	<0.001 ***
COPE (TO)	0.2143	0.0610	3.516	<0.001 ***
COPE (PA)	0.5018	0.0846	5.929	<0.001 ***
COPE (SS)	0.0523	0.0661	0.792	0.428
COPE (OP)	0.2588	0.1008	2.567	0.010 *
R^2^	0.260			
Adj. R^2^	0.255			

## Data Availability

Not applicable.

## Supplementary Materials

The following supporting information can be downloaded at: https://www.mdpi.com/article/10.3390/ejihpe12090093/s1, Supplementary Table S1: Correlations among the employed variables.
