# Supplementary material for: Sociodemographic and Digital Education Factors Are Associated to General Health, Coping Strategies, and Academic Outcomes of Undergraduate Students during the Post-Pandemic Period
Source: Eur J Investig Health Psychol Educ. 2022 Sep 6;12(9):1334–48. doi: 10.3390/ejihpe12090093 (PMC9498009; doi:10.3390/ejihpe12090093)
Supplement: Supplementary file 1 [file ejihpe-12-00093-s001.zip › ejihpe-1853555-supplementary.pdf]

Supplementary Table S1. Correlations among the employed variables. *: p<.05; **: p<.01; ***: p<.001.

	Age	Gen	U-P	N-C	Q-C	EXP	h-pand	h-pre	UCLA (ISO)	UCLA (RC)	UCLA (TL)	COPE (AS)	COPE (TO)	COPE (PA)	COPE (SS)	COPE (OP)	ROL	QOL	A-C	C-C	GHQ
**Age**	—																				
**Gender**	.049	—																			
**University's PPolicies (U-P)**	.217***	-0.046	—																		
**Number of Cohabitants (N-C)**	-.100*	-0.005	0.035	—																	
**Quality of Internet Connection (Q-C)**	.110**	-0.083*	0.049	-0.065	—																
**Expertise in the Use of Devices (EXP)**	.034	-0.000	0.081*	-0.090*	0.232***	—															
**Use of Devices during the Pandemic (h-pand)**	-.151***	-0.058	-0.029	0.091*	-0.032	0.061	—														
**Use of Devices before the Pandemic (h-pre)**	-0.089*	-0.060	0.110**	0.173***	0.022	0.044	0.664***	—													
**Isolation (UCLA ISO)**	-0.028	-0.042	-0.160***	-0.010	-0.095**	-0.063	0.074	0.019	—												
**Rel. Connectedness (UCLA RC)**	0.016	0.030	-0.054	-0.003	-0.098**	-0.041	-0.001	-0.064	0.597***	—											
**Trait Loneliness (UCLA TL)**	-0.110**	0.055	-0.200***	0.053	-0.121**	-0.042	0.017	-0.032	0.486***	0.512***	—										
**Avoiding Strategy (COPE AS)**	-0.032	0.018	0.021	0.065	-0.048	-0.006	0.043	0.053	0.276***	0.200***	0.235***	—									
**Transcendent Orientation (COPE TO)**	0.201***	0.005	0.304***	0.055	0.069	0.033	-0.024	0.078	-0.091*	-0.098*	-0.094*	0.046	—								
**Positive Attitude (COPE PA)**	0.062	-0.034	0.015	0.037	0.106**	0.050	-0.032	0.008	-0.219***	-0.238***	-0.241***	-0.162***	0.092*	—							
**Social Support (COPE SS)**	-0.080*	-0.072	-0.092***	0.039	-0.024	-0.069	-0.010	-0.012	-0.039	-0.325***	-0.154***	0.068	0.105**	0.091*	—						
**Orientation to Problem (COPE OP)**	0.024	-0.064	-0.040	-0.000	0.116**	0.106**	-0.009	-0.025	-0.181***	-0.182***	-0.202***	-0.298***	0.055	0.535***	0.183*	—					
**Resilience to Online Learning (ROL)**	-0.179***	0.066	-0.234***	0.057	-0.024	-0.035	-0.011	-0.084*	-0.014	0.015	0.049	0.028	-0.158***	-0.027	0.043	0.041	—				
**Quality of Online Learning (QOL)**	0.155***	-0.046	0.228***	-0.003	0.197***	0.172***	-0.104**	-0.066	-0.199***	-0.108**	-0.082*	-0.007	0.153***	0.066	-0.010	0.028	0.249***	—			
**Number of Attended Courses (AC)**	-0.106	0.027	-0.161	0.098	0.053	-0.045	0.071	0.168	0.134	0.143	0.151	0.150	0.019	0.004	0.115	0.094	0.182	0.026	—		

**Number of Completed Courses (CC)**	0.080	0.019	-0.106	0.059	0.055	0.009	-0.192	-0.226*	-0.080	-0.055	-0.151	-0.315**	0.050	0.136	-0.054	0.181	0.043	-0.056	-0.012	—	
**General Health (GHQ)**	0.117**	0.075	0.171***	0.002	0.130***	0.126**	-0.089*	-0.017	-0.546***	-0.459***	-0.392***	-0.378***	0.137***	0.361***	0.061	0.342***	-0.096*	0.162***	-0.212***	0.121	—
